# *Lactiplantibacillusplantarum* APsulloc331261 (GTB1^™^) promotes butyrate production to suppress mucin hypersecretion in a murine allergic airway inflammation model

**DOI:** 10.3389/fmicb.2023.1292266

**Published:** 2024-02-21

**Authors:** Hye-Shin Kim, Bobae Kim, Wilhelm H. Holzapfel, Hyeji Kang

**Affiliations:** ^1^Department of Advanced Convergence, Handong Global University, Pohang, Republic of Korea; ^2^HEM Pharma, Pohang, Republic of Korea

**Keywords:** probiotics, SCFA, allergic asthma, mucus hypersecretion, MUC5AC

## Abstract

**Introduction:**

Allergic airway diseases are one of the serious health problems in worldwide and allergic airway inflammation is a prerequisite led to the exacerbated situation such as mucus hypersecretion, epithelial barrier damage and microbiota dysbiosis. Because of side effects and low efficiencies of current therapeutics, the need for novel alternatives has been urged. Probiotics in which have diverse and beneficial modulatory effects have been applied to the airway inflammation model and the underlying mechanism needs to be investigated.

**Methods:**

We aimed to evaluate whether our target strain, Lactiplantibacillus plantarum APsulloc331261 (GTB1TM) isolated from green tea, can ameliorate allergic airway inflammation in mice and to figure out the mechanism. We induced allergic airway inflammation to mice by ovalbumin (OVA) and administered GTB1 orally and the immune and epithelial barrier markers were assessed. The gut metabolite and microbiota were also analysed, and the in vitro cell-line experiment was introduced to confirm the hypothesis of the study.

**Results:**

GTB1 ameliorated type 2 inflammation and suppressed mucin hypersecretion with the inhibition of MUC5AC in inflamed mice. Moreover, GTB1 increased the butyrate production and the relative abundance of butyrate producer, Clostridium cluster IV. We assumed that butyrate may have a potential role and investigated the effect of butyrate in mucin regulation via human airway epithelial cell line, A549. Butyrate significantly reduced the gene expression of MUC5AC in A549 cells suggesting its regulatory role in mucus production.

**Conclusion:**

Therefore, our study demonstrates that the oral administration of GTB1 can ameliorate allergic airway inflammation and mucin hypersecretion by butyrate production.

## 1 Introduction

Asthma is one of the most prevalent chronic respiratory diseases and is considered a serious health problem affecting more than 339 million people in the world (The Global Asthma Report, 2018). It has been demonstrated as a more complicated disease than the previous paradigm because of its heterogeneity attributed to genetic and epigenetic variability of individuals, and the cooperation of environmental exposures. This influence connects to various diversities and severities of the disease (Carr and Bleecker, 2016). The underlying disease development processes, clinical symptoms, and reactions to medications are diverse due to their heterogeneity; therefore, the specific approach depending on the subtypes is important (Global Initiative for Asthma, 2021).

The most common phenotype, allergic asthma, is driven by the sensitization and challenge phase against repetitive stimuli and is possessed by more than 80% of children and most adults with asthma, suggesting its seriousness and necessity for the exploration of novel therapeutics (Holgate et al., 2015). Type 2 inflammation is frequently observed in this subtype and is considered a prerequisite for the development and exacerbation of the diseased state. In this reaction, type 2 helper T cell (Th2) plays a central role by secreting related cytokines such as interleukin (IL)-4 and IL-5. Th2 cytokines activate plasma B cells to produce immunoglobulin E (IgE) recruiting more downstream effector cells such as eosinophils, basophils, and mast cells with the secretion of pro-inflammatory mediators (Fahy, 2015).

The amplified cascade of inflammation is related to airway remodeling. As the severity and duration of the disease increase, the thickness of airway smooth muscle increases and the goblet cells overproduce mucus to form a plug that can induce airway hyperresponsiveness and air trapping (Holgate et al., 2015). Particularly, overexpressed or altered goblet cells, called goblet cell hyperplasia and metaplasia, are eventually representative of airway obstruction. Mucus hypersecretion is associated with the excess upregulation of specific secretory mucins, such as Mucin 5 AC (MUC5AC) and Mucin 5B (MUC5B), and its related signaling marker such as calcium-activated chloride channel regulator 1 (CLCA1). These markers increase under asthmatic conditions proving their pathogenic role in allergic asthma (Evans et al., 2015; Bonser and Erle, 2017; Liu and Shi, 2019). Therefore, to keep the balance in the mucosal layer, its protective role in immunity should be sustained properly.

Probiotics have been studied for their potential use as an alternative remedy for allergic asthma, based on their modulatory effects on the immune system and mucosal barrier (Martens et al., 2018). *Lactobacilli* are the most widely represented group of lactic acid bacteria (LAB) prevalently used for probiotics due to their diverse beneficial functions and their potential for combating a variety of inflammatory diseases including asthma (Klein et al., 1998; Mortaz et al., 2013). Hong et al. (2010) reported that several heat-killed *Lactobacillus* species alleviated allergic asthmatic reactions in mice models with the suppression of inflammatory markers and mucin markers including MUC5AC and mouse homolog of CLCA1 (mCLCA1). Anatriello et al. (2019) found *L. bulgaricus* strain can attenuate lung inflammation and mucus overproduction by modulating T-cell responses. This former background research revealed that putative probiotics can alleviate allergic asthmatic reactions by regulating not only immune markers but also epithelial markers, especially those related to mucin production.

The concept of the gut–lung axis, the cross-talk between the gastrointestinal tract (GIT) and respiratory tract, has emerged to explain the underlying ameliorating mechanisms derived from LAB in the diseased state (Dang and Marsland, 2019). Both gut and lungs are mucosal organs and accommodate diverse microbial communities affecting host health in a variety of ways including immune maturation, epithelial barrier fortification, and production of beneficial metabolites (Budden et al., 2017; Eshleman and Alenghat, 2021; Wang et al., 2021). Short-chain fatty acids (SCFAs) belong to the major microbial metabolites produced from indigestible fiber and have been considered important key mediators in respiratory diseases in a specific airway inflammation model. Based on the study of Trompette et al. (2014), SCFA produced by the consumption of a high-fiber diet elicited a protective effect against allergic airway inflammation with the elevation of serum SCFA level in a murine model. Moreover, the supplementation of SCFA in mice decreased the susceptibility to asthma by modulation of T-cell response, while the single administration of butyrate directly restrained Th2 cell polarization (Cait et al., 2018). Subsequently, mice fed a high-fiber diet showed increased SCFA levels concomitantly with the inhibition of airway hyperresponsiveness and airway inflammation (Lewis et al., 2019). Similarly, the versatility and efficacy of SCFA in airway inflammation have been investigated extensively. However, the exact underlying mechanisms need to be explored further.

*Lactiplantibacillus plantarum* is one of the *Lactobacillus* species with the largest genomes because its versatility has been extensively explored for combating a wide spectrum of diseases, especially intestinal inflammatory disorders (Yokota et al., 2018; Yu et al., 2020; Ding et al., 2021). This scope has been expanded to include allergic inflammatory disease models aiming at sites distal to the gut such as the upper respiratory tract (Choi et al., 2018; Kim et al., 2019). Several research studies focused on the effect of *L. plantarum* strains in the lower respiratory tract, using trials with diverse administration schemes and airway inflammation models (Pellaton et al., 2012; Liu et al., 2014; Jin et al., 2020). Previously, we investigated GTB1, isolated from green tea by Amorepacific Corporation (Seoul, Republic of Korea), and reported on its genomic information and demonstrated its safety (Arellano et al., 2020). Moreover, it was reported that GTB1 alleviated gastric inflammation with the alteration of SCFA production and gut microbiota (Park et al., 2020).

Hence, we aimed to explore whether GTB1 ameliorates allergic asthma in a murine model by focusing on SCFA production and highlight the potential of this strain as a putative probiotic.

## 2 Materials and methods

### 2.1 Bacterial preparation

GTB1 (deposit number: KCCM11179P, GTB1^™^) was provided by the Amorepacific R&I Center (Republic of Korea). The strain was grown in de Man, Rogosa and Sharpe (MRS) (Becton, Dickinson and Company (BD), Franklin Lakes, NJ, United States) medium and incubated under anaerobic conditions at 37°C for 16 h. The cultured bacteria were washed two times and used for the experiment. The washing protocol used in this study consisted of several steps: First, the bacterial culture was centrifuged at 3,000 × g for 15 min at 4°C to pellet the cells. After discarding the supernatant, an equivalent volume of sterile 1X PBS was added, followed by thorough vortexing. This process was repeated, with additional centrifugation and discarding of the supernatant. The final step involved adding an equivalent volume of sterile 1X PBS to prepare the bacterial suspension for oral gavage to achieve the desired volume. For heat-killed bacterial preparation, the bacterial suspension with a desired dose was heat-killed for 15 min at 80°C. After heat treatment, the viability of the bacterial suspension was checked by spreading on MRS agar.

### 2.2 OVA-induced allergic airway inflammation murine model and bacterial treatment

Six-week-old female BALB/c mice were housed in specific pathogen-free conditions. The allergic airway inflammation murine model followed the research of Reber et al. (2012) with some modifications. Mice were sensitized with 50 μg of OVA (Grade V) (Sigma-Aldrich, Saint Louis, MO, United States) with 2 mg of aluminum hydroxide (alum) (Sigma-Aldrich, United States) on days 0 and 7 by intraperitoneal injection. After the last sensitization, mice were intranasally challenged with 10 μg of OVA on days 14, 17, 21, 24, 28, and 31. Bacterial administration (live or heat-killed) was carried out over 2 weeks, with the treatment given for 5 consecutive days each week, followed by euthanization during the last week after 4 days of treatment. The administration was done via oral (1 × 10^9^ CFU/mouse) or intranasal (1 × 10^7^ CFU/mouse) routes. On day 32, mice were euthanized, and the serum, bronchoalveolar lavage fluid (BALF), lung, colon, and feces were collected for further analysis. All intranasal injections and euthanization were conducted after anesthesia with an intraperitoneal injection of ketamine/xylazine mixture [ketamine (64 mg/kg; Yuhan ketamine injection, Yuhan Corporation, Seoul, Republic of Korea); xylazine (13 mg/kg; Rompun, Elanco Korea, Ansan, Republic of Korea)].

### 2.3 Lung histology

Tissues of the mouse lung were fixed in 10% formaldehyde solution for histological analysis including hematoxylin and eosin (H&E), periodic acid–Schiff (PAS), and immunohistochemical staining (IHC), which is specific to mouse monoclonal anti-MUC5AC (sc-21701) (Santa Cruz Biotechnology, Dallas, TX, United States), by KP&T company (Cheongju, Republic of Korea) as a commercial service.

### 2.4 BALF cytokine analysis

BALF supernatant collection was performed based on the protocol of Van Hoecke et al. (2017). BALFs were collected by tracheal infusions of 1 mL phosphate-buffered saline (PBS), and perfused BALFs were centrifuged for 4 min at 700 × g. The supernatants were harvested and stored at −80°C until analysis. The protein levels of IL-4 and IL-5 were measured using the ELISA MAX^™^ Deluxe Set Mouse IL-4 and IL-5 (BioLegend, Inc., San Diego, CA, United States) according to the manufacturer’s protocol.

### 2.5 Serum immunoglobulin analysis

Blood was collected and centrifuged at 1,500 × *g* for 20 min. The obtained serum was stored at −80°C for the immunoglobulin analysis. The level of total IgE in diluted serum was measured using the ELISA MAX^™^ Deluxe Set Mouse IgE (BioLegend) kit. The level of OVA-specific IgE was analyzed using the LEGEND MAX^™^ Mouse OVA Specific IgE ELISA Kit (BioLegend). Every assay was performed according to the manufacturer’s instructions.

### 2.6 Messenger RNA extraction and quantitative real-time PCR analysis

The mRNA was extracted from the collected tissues with TRIzol (Invitrogen^™^, Carlsbad, CA, United States) following the manufacturer’s protocol. The extracted mRNA was reverse-transcribed into complementary DNA with a GoScript^™^ Reverse Transcription kit (Promega, Madison, WI, United States). A quantitative real-time PCR was performed using the TB Green^™^ Premix Ex Taq kit (Takara Bio, Shiga, Japan) and the Step-One Plus real-time PCR system (Applied Biosystems, Walsum, MA, United States). Data were quantified using the comparative threshold cycle method. Actin beta (ACTB) was used for housekeeping gene control. The primer information used in this research is provided in Supplementary Table S1.

### 2.7 SCFA analysis

The SCFA levels in the cecum and feces were analyzed as a commercial service by HEM Pharma Inc. (Gwanggyo, Republic of Korea). All SCFAs were extracted with 0.1 g of mouse fecal sample in 1 mL of distilled water. Vortexed samples were centrifuged, the supernatant was transferred to a vial with GC buffer solution, and 2-ethylbutric acid was added as an internal standard (Zhang et al., 2018). An HSS-GC-FID analysis was conducted using the Agilent 7890B GC system with a 7697A headspace sampler and FID (Agilent Technologies, US). ChemStation software (Agilent Technologies) was used for data acquisition and processing. SCFAs were identified and quantified using standard compounds, such as acetic acid, propionic acid, butyric acid, iso-butyric acid, iso-valeric acid, and valeric acid (Sigma-Aldrich).

### 2.8 DNA extraction and next-generation sequencing analysis

DNA extraction and NGS analysis were conducted by HEM Pharma Inc. In brief, extracted bacterial DNA in feces was analyzed regarding the variable V3–V4 regions of the 16S ribosomal RNA gene. The V3–V4 region was amplified using the 341F (5′-TCGTCGGCAGCGTCAGATGTGTATAAGAGACAGCCTACGGGNGGCWGCAG-3′) and 805R (5′- GTCTCGTGGGCTCGGAGATGTGTATAAGAGACAGGACTACHVGGGTATCTAATCC-3′) primers. Sequencing was performed using the Illumina MiSeq System (Illumina). The concentration of libraries was verified and sequenced using the Illumina MiSeq System. Reads were sorted using the unique barcodes for each PCR product. The barcode, linker, and primer sequences were then eliminated from the original sequencing reads for the next analysis step.

### 2.9 Bioinformatic analysis

Single-end FASTQ files were imported into the Quantitative Insights of Microbial Ecology 2 (QIIME2, ver. 2021.2) via the FASTQ manifest protocol (Caporaso et al., 2010; Bolyen et al., 2019). Denoising was conducted with DADA2 (Callahan et al., 2016). MAFFT plugin was used for the alignment of all amplicon sequence variants (ASVs), and a phylogeny analysis was determined with fasttree2 based on the Greengenes database (Price et al., 2010; Katoh and Standley, 2013). All sampling depths reached 19,400 reads. Alpha diversity metrics were analyzed using the Shannon index (Shannon and Weaver, 1949), Simpson index (Simpson, 1949), and Chao1 index (Chao, 1984). Beta diversity was analyzed with the Bray–Curtis dissimilarity method (Sorenson, 1948), and a principal coordinate analysis (PCoA) was performed to evaluate the microbial distances between the groups.

### 2.10 Cell cultivation and butyrate treatment

The human airway epithelial cells (A549, distributed by the Korean Cell Line Bank) were grown in RPMI 1640 media (Gibco^™^, Thermo Fisher Scientific, Waltham, MA, United States) containing 10% fetal bovine serum (Gibco^™^), 1% antibiotics (antibiotic-antimycotic, Gibco^™^), and 1% non-essential amino acids solution (Gibco^™^) at 36.5°C and 5% CO_2_ condition. The cells were seeded into 96-well plates (Corning^®^, New York, NY, United States) with non-supplemented cell media and maintained until confluency. The grown cells were incubated with different concentrations of butyrate (0.1 mM and 2 mM) supplemented media for 4 h. The control group was treated with vehicle-supplemented media. All cells were collected to measure the gene expression of MUC5AC.

### 2.11 Statistical analysis

Data were analyzed by GraphPad Prism software 7.0 (GraphPad Software, La Jolla, CA, United States) and expressed as the mean ± SD. Statistical significance was calculated using the one-way ANOVA with Fisher’s LSD test. *p*-values of less than 0.05 were considered statistically significant.

## 3 Results

### 3.1 Effects of GTB1 on allergic airway inflammation in mice

**Figure 1:**
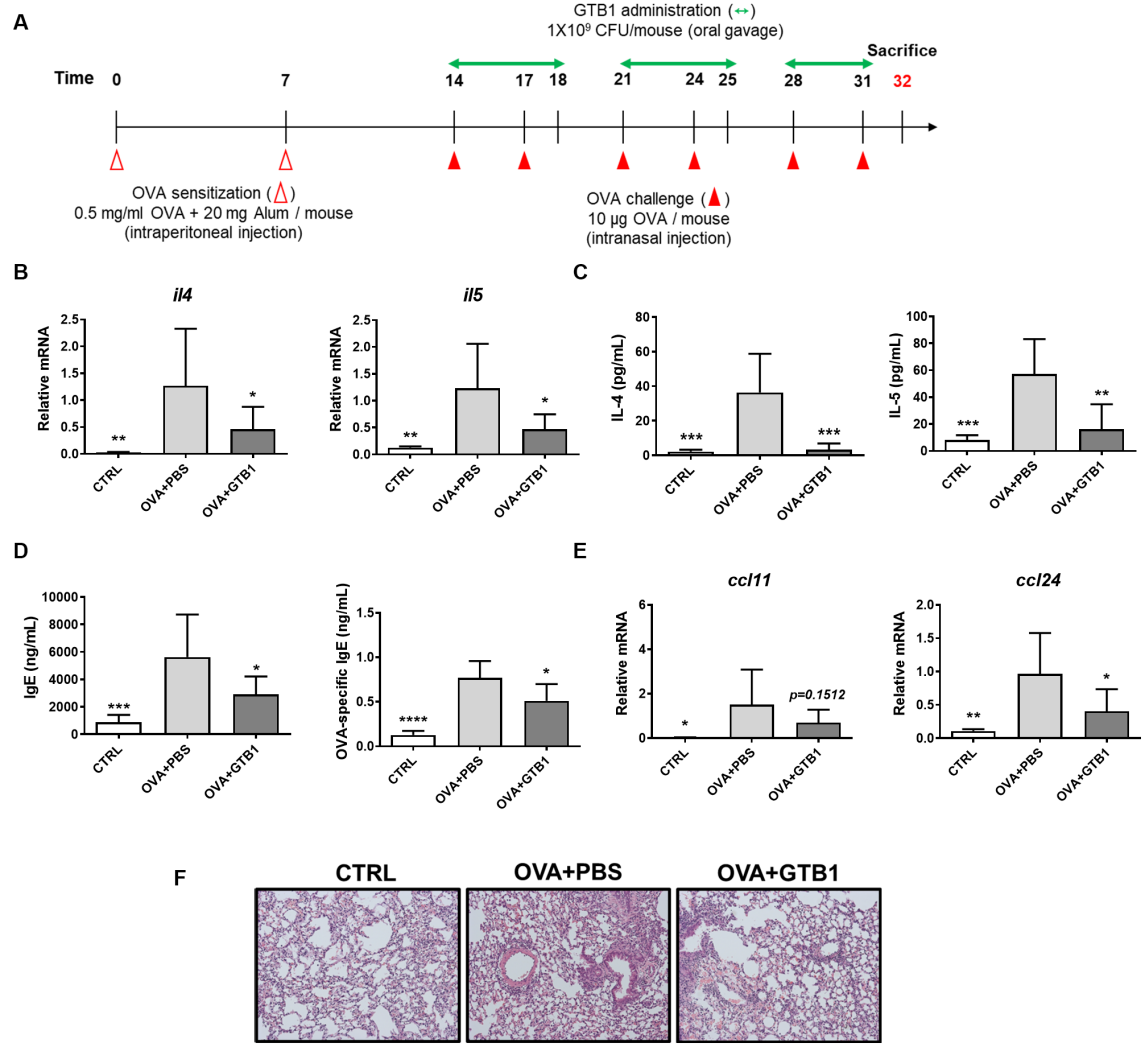
Effects of oral administration of GTB1 on type 2 inflammation in an OVA-induced allergic airway inflammation murine model. **(A)** Scheme of the *in vivo* experiment, **(B)** lung mRNA expression levels of type 2 cytokines, **(C)** Th2 cytokines in BALF, **(D)** total IgE and OVA-specific IgE level in serum, **(E)** lung mRNA expression of type 2 chemokines, and **(F)** representative images of H&E-stained lung tissue in each group (magnification: 100X). *Il4*: IL-4; *Il5*: IL-5; *Ccl11*: C-C motif chemokine ligand (CCL)-11; *Ccl24*: CCL24; CTRL: control group treated with vehicle only; OVA + PBS: OVA + vehicle-treated group; OVA + GTB1: OVA + GTB1-treated group. Data show the mean ± SD (*n* = 5–7 mice per group); the difference compared to the OVA + PBS group was analyzed using the one-way ANOVA with Fisher’s LSD test. *p < 0.05, **p < 0.01, ***p < 0.001, and ****p < 0.0001.

We evaluated whether GTB1 ameliorates allergic airway inflammation, especially regarding type 2 inflammation, in a murine model. Mice were sensitized and challenged by OVA allergen to induce allergic airway inflammation, and GTB1 was orally administered according to the experimental scheme (Figure 1A). The levels of cytokine, immunoglobulin, and chemokine markers were determined using qRT-PCR or ELISA (Figures 1B–E). Oral administration of our target strain significantly reduced the transcript and protein levels of type 2 inflammatory cytokines such as IL-4 and IL-5 (Figures 1B,C). Total IgE and OVA-specific IgE levels in the serum were also significantly decreased in the GTB1 group compared to the OVA + PBS group (Figure 1D). Additionally, the gene expression of the chemokine related to type 2 inflammation, CCL24, significantly decreased in the GTB1-treated group, and there was a tendency for CCL11 to decrease as well. The inflammatory cell infiltration and thickness of smooth muscle around lung alveoli were improved in our target strain-administered group (Figure 1F). Collectively, oral application of GTB1 attenuates allergic airway inflammation. Unlike the results of oral administration, intranasal treatment of the strain could not suppress the type 2 inflammation markers and even increased the levels of IgE in the serum (Supplementary Figure S1). We attempted to evaluate the efficacy of GTB1 after heat inactivation. Heat-killed GTB1 decreased the level of IL-4 on the protein level but failed to significantly suppress IL-5 and IgE (Supplementary Figure S2). These results suggest oral administration of live GTB1 to be the most effective for ameliorating allergic airway inflammation in mice.

### 3.2 Effects of GTB1 on MUC5AC and mucin hypersecretion in the lungs

**Figure 2:**
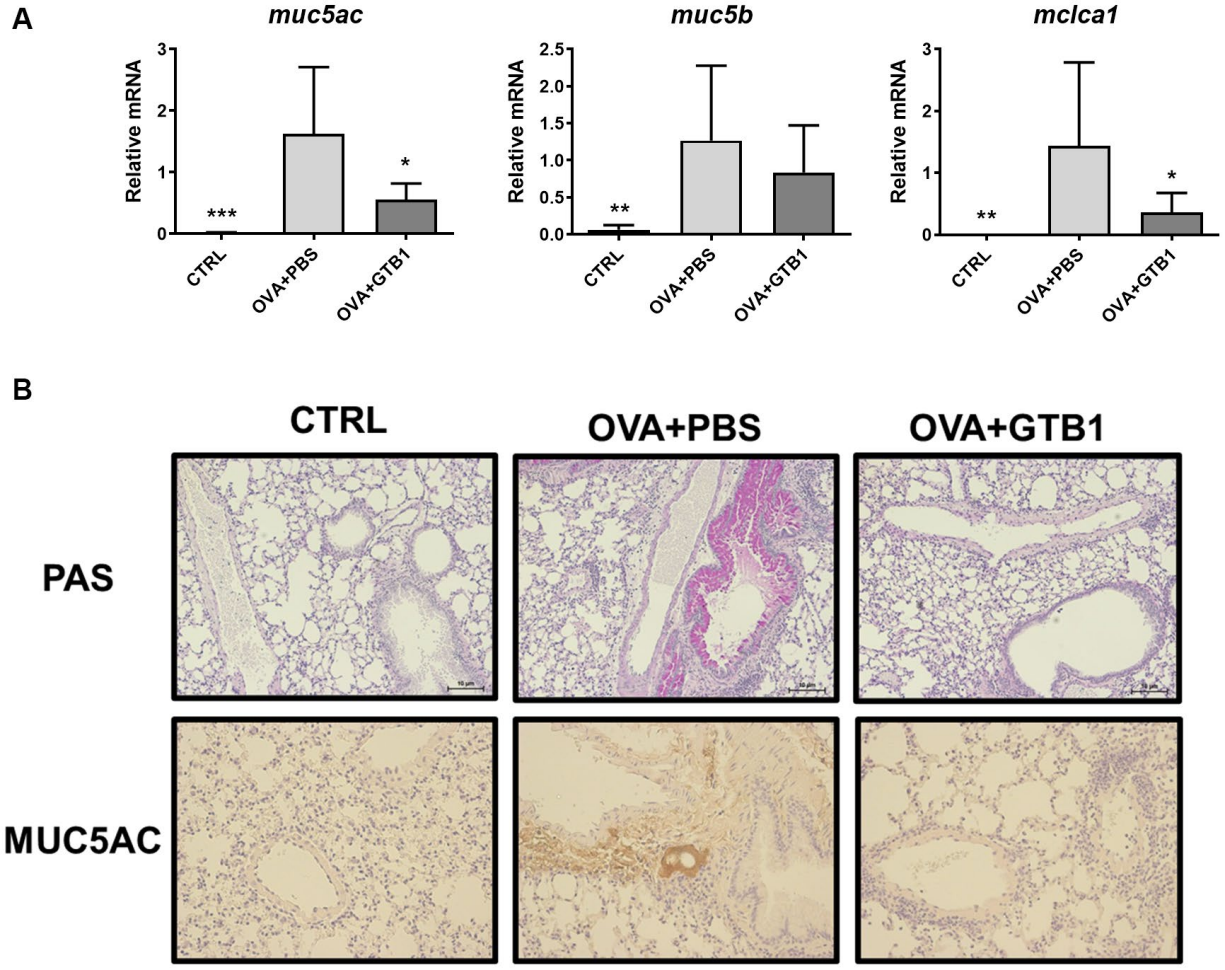
Effects of oral administration of GTB1 on MUC5AC production and mucin hypersecretion in an OVA-induced allergic airway inflammation murine model. **(A)** Lung mRNA expression level of MUC5AC signaling markers including secretory mucins (MUC5AC and MUC5B) and mouse homolog of channel protein for mucin secretion (CLCA1) and **(B)** the representative images of Periodic acid–Schiff (PAS)-stained and anti-MUC5AC stained lung tissue of each group (100x magnification). *Muc5ac*: MUC5AC; *Muc5b*: MUC5B; *Clca1*: mouse homolog of calcium-activated chloride channel regulator 1; CTRL: control group treated with vehicle only; OVA + PBS: OVA + vehicle-treated group; OVA + GTB1: OVA + GTB1-treated group. Data show the mean ± SD (*n* = 5–7 mice per group); the difference compared to the OVA + PBS group was analyzed using the one-way ANOVA with Fisher’s LSD test. *p < 0.05, **p < 0.01, and ***p < 0.001.

Furthermore, the gene expression of secretory mucins (MUC5AC and MUC5B) and intermediate channel protein, CLCA1, was measured to determine the effects of oral administration of live GTB1 on the airway epithelial barrier, specifically on mucin hypersecretion. Oral administration of live GTB1 successfully downregulated the gene expression of MUC5AC and its dependent protein CLCA1 in the lungs (Figure 2A). PAS staining and anti-MUC5AC immunohistochemical staining of mouse lung sections confirmed the reduction of total mucin and MUC5AC production and demonstrated the decreased mucin hypersecretion by GTB1 (Figure 2B). Comparatively, the gene expression of tight junction proteins was not improved in spite of the treatment of the target strain (Supplementary Figure S3). These data show that GTB1 may inhibit MUC5AC hypersecretion, thereby resulting in the inhibition of mucus hypersecretion.

### 3.3 Butyrate production of GTB1 in the gut

**Figure 3:**
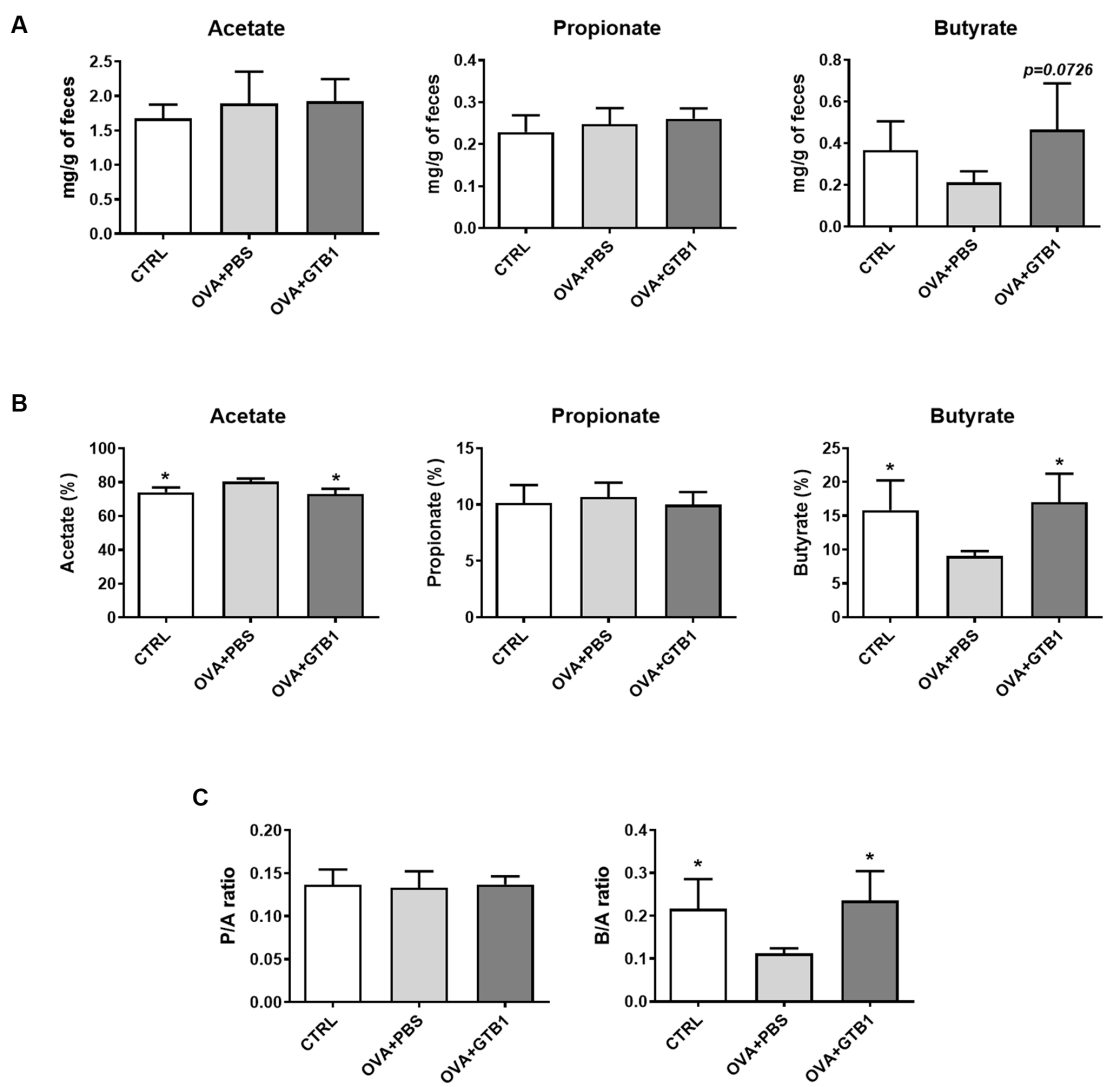
Effects of oral administration of GTB1 on fecal SCFA production in an OVA-induced allergic airway inflammation murine model. **(A)** Absolute concentrations of the three main SCFAs, **(B)** relative percentage of each SCFA, and **(C)** the ratio of propionate/acetate and butyrate/acetate. CTRL: control group treated with vehicle only; OVA + PBS: OVA + vehicle-treated group; OVA + GTB1: OVA + GTB1-treated group. Data show the mean ± SD (*n* = 3–5 mice per group); the difference compared to the OVA + PBS group was analyzed using the one-way ANOVA with Fisher’s LSD test. *p < 0.05.

The levels of representative SCFA were measured in cecum and feces to explain how oral administration of GTB1 may affect local immune reactions. While no significant alteration in acetate and propionate was detected in the group of GTB1, the absolute and relative levels of butyrate increased in the feces of the target strain-treated group compared to the OVA + PBS group (Figure 3). Moreover, the consumption of GTB1 also elevated the ratio of butyrate/acetate (Figure 3C). The level of butyrate in the cecum was increased significantly compared to the OVA + PBS group; on the other hand, no remarkable alteration in acetate and propionate levels could be detected in the treated group (Supplementary Figure S4). These results suggest that oral administration of GTB1 promoted butyrate production in the gut.

### 3.4 Gut microbiota modulation by GTB1

**Figure 4:**
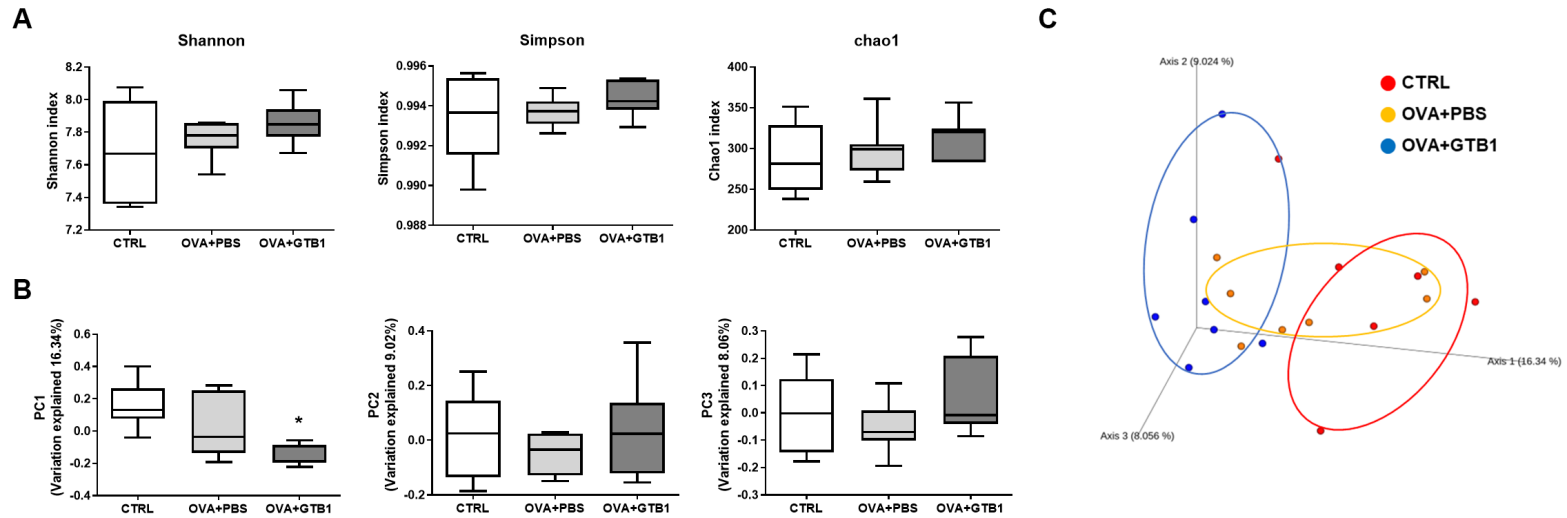
Effects of oral administration of GTB1 on gut microbiota diversity in an OVA-induced allergic airway inflammation murine model. **(A)** Alpha diversity indices (from left; Shannon, Simpson, and Chao1), **(B)** principal coordination analysis (PCoA) plot based on Bray–Curtis distance, and **(C)** PC values of Bray–Curtis distance analysis. CTRL: control group treated with vehicle only; OVA + PBS: OVA + vehicle-treated group; OVA + GTB1: OVA + GTB1-treated group. Data show the mean ± SD (*n* = 6–7 mice per group); the difference compared to the OVA + PBS group was analyzed using the one-way ANOVA with Fisher’s LSD test. *p < 0.05.

**Figure 5:**
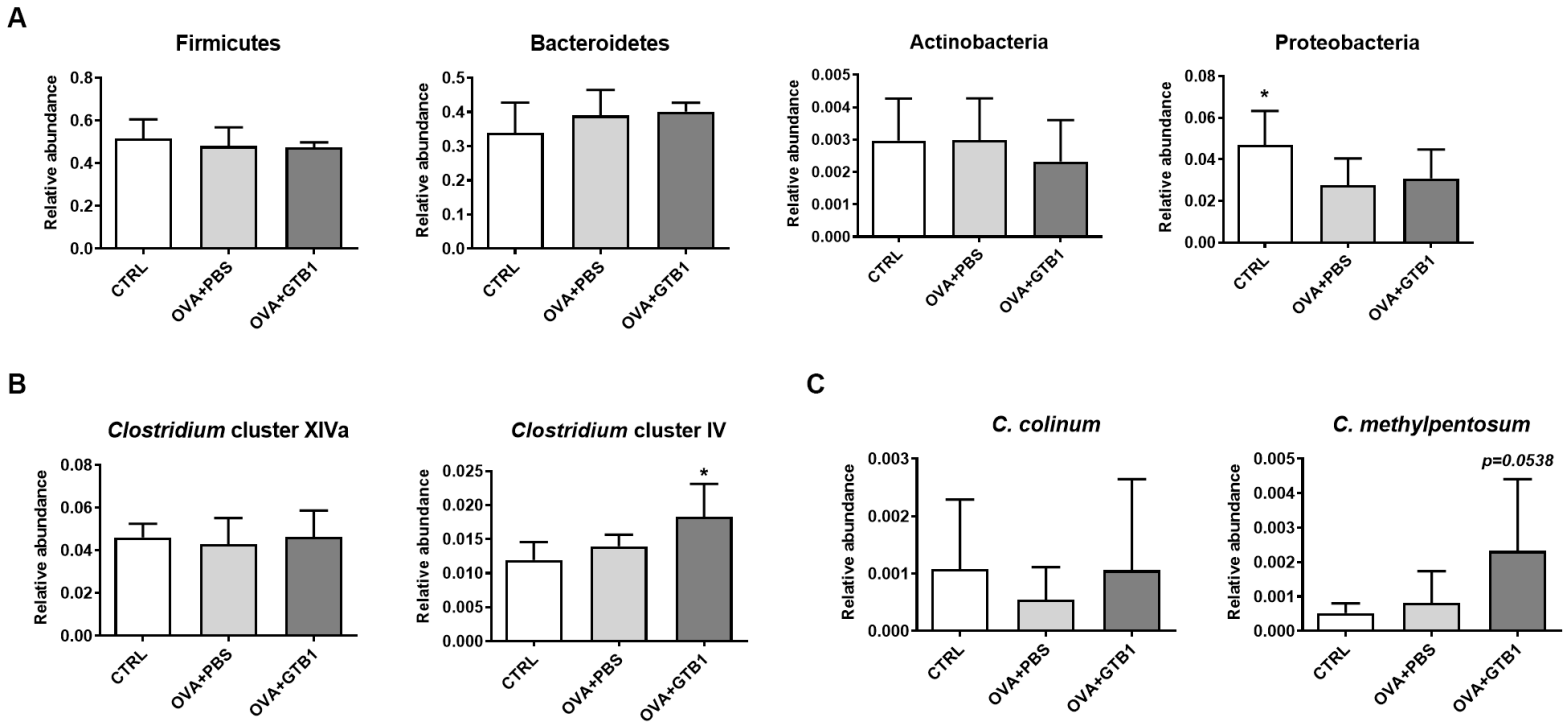
Effects of oral administration of GTB1 on gut microbiota composition in an OVA-induced allergic airway inflammation murine model. The levels of relative abundance in **(A)** phyla, **(B)** genus, and **(C)** species. CTRL: control group treated with vehicle only; OVA + PBS: OVA + vehicle-treated group; OVA + GTB1: OVA + GTB1-treated group. Data show the mean ± SD (*n* = 6–7 mice per group); the difference compared to the OVA + PBS group was analyzed using the one-way ANOVA with Fisher’s LSD test. *p < 0.05.

To determine whether GTB1 alters the gut microbiota, gut microbiota diversity and composition were analyzed. The Shannon, Simpson, and Chao1 indices were used for the analysis of alpha diversity. The results revealed that GTB1 did not have any significant impact on the richness or evenness (Figure 4A). However, the beta diversity was determined by the Bray–Curtis distance metric analysis, and the GTB1-treated mice showed a significant difference in the PC1 value compared to the OVA + PBS group and also displayed a clear clustering in the PCoA plot (Figures 4B,C). In taxonomical analysis, while there was no significant difference between the groups in the phylum level (Figure 5A), an alteration was observed at the genus and the species levels. The relative abundance of *Clostridium* cluster IV was notably increased in the target strain-treated group, while that of *Clostridium* cluster XIVa was not altered in this group (Figure 5B). On the species level, the relative abundance of *C. colinum*, a species of *Clostridium* cluster XIVa detected in the *in vivo* sample, did not increase markedly (*p* = 0.4342). However, the relative abundance levels of *C. methylpentosum*, belonging to *Clostridium* cluster IV, were increased (*p* = 0.0538) after GTB1 treatment (Figure 5C).

### 3.5 Impact of GTB1 on SCFA uptake receptor expression

**Figure 6:**
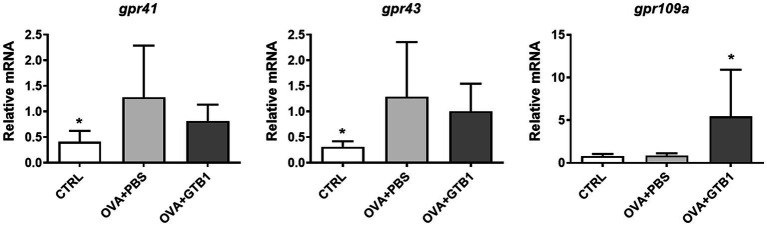
Effects of oral administration of GTB1 on modulation of the gene expression of SCFA uptake G protein-coupled receptors in the lungs. CTRL: control group treated with vehicle only; OVA + PBS: OVA + vehicle-treated group; OVA + GTB1: OVA + GTB1-treated group. Data show the mean ± SD (*n* = 6–7 mice per group); the difference compared to the OVA + PBS group was analyzed using the one-way ANOVA with Fisher’s LSD test. *p < 0.05.

To determine whether the increased butyrate is absorbed in the target site of the diseased condition, the gene expression of G protein-coupled receptor (GPR)-related SCFA uptake was measured in the lungs. Oral administration of GTB1 significantly upregulated mRNA expression of GPR109a which is responsible for butyrate uptake (Figure 6). These data suggest that GTB1 modulated the gene expression of butyrate uptake receptors in the lungs.

### 3.6 Suppression of MUC5AC mRNA expression by butyrate in human airway epithelial cells *in vitro*

**Figure 7:**
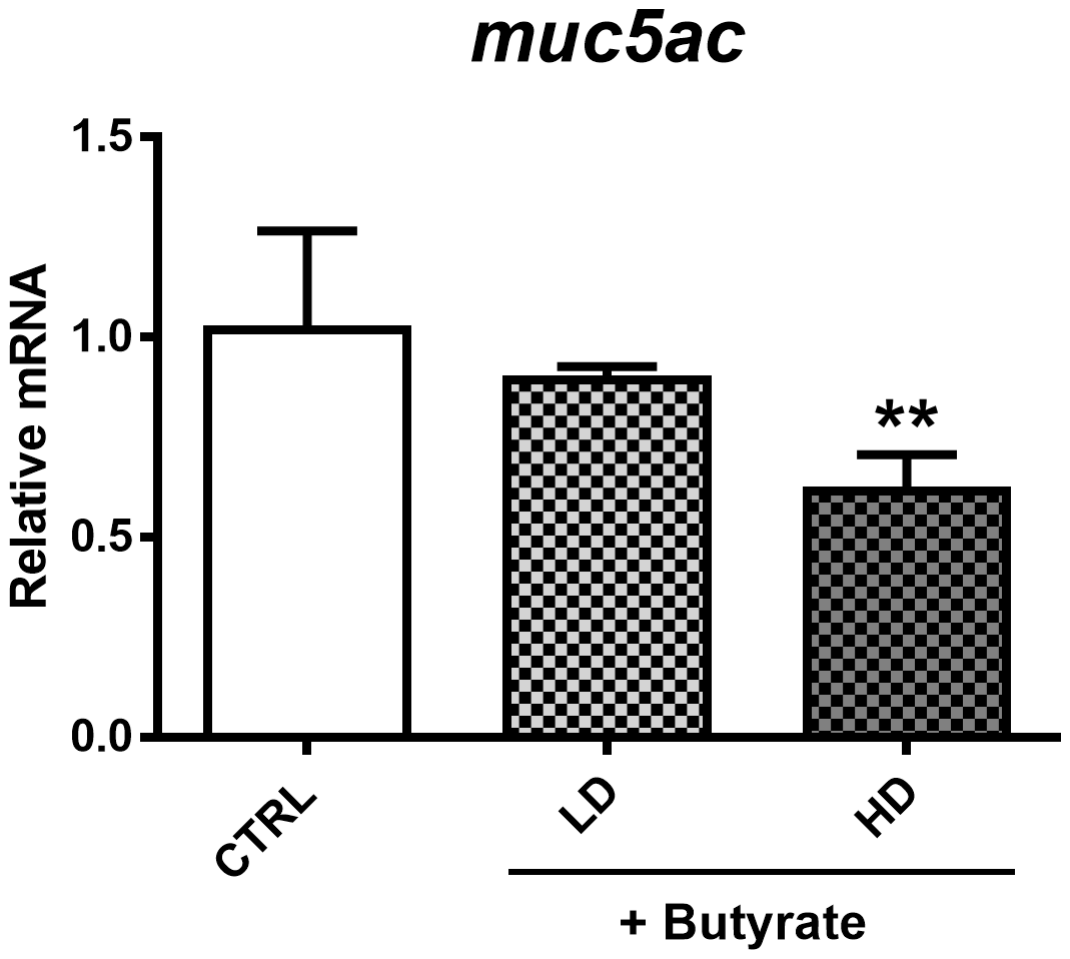
Modulation of MUC5AC mRNA expression by butyrate in A549 human airway epithelial cells. After confluency, low (0.1 mM) and high (2 mM) concentrations of butyrate were incubated with A549 cells for 4 h, and MUC5AC gene expression was measured from harvested cell mRNA. CTRL: vehicle only treated control group; LD: low dose group; HD: high dose group. Data show the mean ± SD (*n* = 3); the difference compared to the CTRL group was analyzed using the one-way ANOVA with Fisher’s LSD test. *p < 0.05 and **p < 0.01.

As shown before, GTB1 ameliorated allergic airway inflammation and mucus hypersecretion along with gut microbiota modulation and butyrate production. Based on the elevated gene expression of the butyrate uptake receptor, we exposed A549 human airway epithelial cells to butyrate to investigate the direct influence of butyrate on mucus hyperproduction *in vitro*. After co-incubation with butyrate and cells, MUC5AC gene expression was measured. Butyrate inhibited the gene expression of MUC5AC in A549 cells in a dose-dependent manner suggesting its direct and inhibitory effects on MUC5AC expression in airway epithelial cells (Figure 7).

## 4 Discussion

Allergic asthma is a chronic inflammatory disease that is characterized by allergic airway inflammation and airway remodeling, eventually resulting in the malfunction of the respiratory tract (Murdoch and Lloyd, 2010). Airway remodeling designates structural alterations in the airway wall. These include the thickening of the smooth muscle of the airway wall, infraction of the barrier, and abnormal increase of goblet cells (Lambrecht and Hammad, 2012). Airway remodeling is highly relevant to airway mucus hypersecretion, and it contributes to airway obstruction and increasing morbidity and mortality (Rogers, 2004). Nonetheless, a pharmaceutical approach was focused on the suppression of inflammation despite the insensitivity to current therapy (Papi et al., 2020). This has widened the scope for a highly required novel alternative that can alleviate dysregulated reactions of the immune system and epithelial lining.

The application of probiotics against allergic asthma has been suggested as a promising alternative due to their multifaceted advantages. LAB are powerful candidates as putative probiotics for the airways. *L. plantarum* is a well-known and widely implemented species, largely used because of its safety, its adaptation to diverse niches, and its beneficial properties. Yet, its application in the therapeutic treatment of allergic asthma is still limited. Based on former studies, we expected GTB1 to have great potential for airway application. According to Arellano et al. (2020), safety assessments regarding hemolysis, biogenic amine production, and antibiotic resistance have been performed for GTB1, and the target strain showed negative results throughout. Moreover, its survivability and adaptability to GIT have been investigated, and it showed higher levels than the *L. plantarum* control strain. These data suggest the superior functionality of GTB1 in the GIT for oral consumption and indicate its potential as a putative probiotic strain. Furthermore, the tested strain showed advantageous effects in a gastric inflammation model with an increase in SCFA production (Park et al., 2020). Therefore, we hypothesize that GTB1 may improve other inflammatory responses by modulation of gut metabolites.

In this study, we investigated the modulatory effects of GTB1 on allergic asthma to find information on the underlying mechanism. The application of this strain to the respiratory tract is a first trial conferring the novelty of this project. GTB1 was administered to mice-induced airway inflammation. PBS was used as the control in this study to eliminate potential media-related effects (Cheng et al., 2022; Bu et al., 2023; Duan et al., 2023). In our results, oral administration of the strain suppressed type 2 inflammation markers systemically (IgE in serum) and locally (type 2 cytokines and chemokines in BALF and lungs) proving that our target strain modulated the dysregulated immune system in an allergic airway inflammation model. Interestingly, the administration route and bacterial viable conditions influenced the efficacy. We attempted three different schemes of administration: (1) oral treatment with live strain, (2) intranasal treatment with the live strain, and (3) oral treatment with the heat-killed strain. Eventually, oral administration with live strain showed greater efficacy with significant improvements as reflected by various markers. Since oral administration of the strain fed was effective, we assume that the effect of GTB1 may be exerted indirectly and by stimulating specific members of the core gut microbiota such as butyric acid producers.

SCFAs are the end products of dietary fiber fermentation by the intestinal microbiota and the most well-known bacterial metabolites with various health benefits by entering the systemic circulation and other tissues. As a result, SCFAs are frequently considered crucial mediators in gut-to-other-organ communication. Altered SCFA production has been demonstrated in various diseases. Several studies demonstrate that butyrate plays a crucial role in modulating both the immune system and the microbial community. This effect has been observed in an allergic airway inflammation model (Cait et al., 2018; Theiler et al., 2019; Yip et al., 2021). Clinical studies have also demonstrated that a high level of butyrate is associated with protection from allergic asthma, while loss of butyrate can increase the risk of asthma development (Chiu et al., 2019; Roduit et al., 2019). We found that the level of butyrate was elevated in the cecal and fecal samples of the GTB1-treated mice (Figure 3, Supplementary Figure S4). These data give an indication that butyrate may play a key role in suppressing allergic airway inflammation through the consumption of GTB1. In diversity analysis, beta diversity (Bray–Curtis distance) between groups showed a clear difference compared to the inflamed group (Figures 4B,C), indicating modulation of microbiota. Taxonomical analysis did not show any notable differences at the phylum level, yet the relative abundance of *Clostridium* cluster IV was significantly increased (Figure 5B). *Clostridium* species are major habitants of the gut accounting for nearly 40% of the total bacteria and are reported as strong producers of butyrate, reported for its regulatory influence on the host immune system (Guo et al., 2020). The reduction of *Clostridium* levels in the intestinal tract of diseased patients has been frequently observed, particularly for *Clostridium* clusters IV and XIVa (Sokol et al., 2009; Zhang et al., 2017). In our study, *Clostridium* cluster XIVa did not show any significant difference between the groups; however, the levels of *Clostridium* cluster IV were more strongly increased than other groups (Figure 5B). At the species level, the relative abundance of *C. methylpentosum* was increased in the GTB1 group (Figure 5C), suggesting that this species is a possible bacterial component responsible for the increase of the butyric acid levels. *C. methylpentosum* was newly identified in 1989 proposing its possible role in intestinal digestion by metabolizing rhamnose (Himelbloom and Canale-Parola, 1989). However, only sparse information is available on possible *in vivo* beneficial effects of *C. methylpentosum*, and more research is needed to clarify this issue. Yet, based on the data obtained in this model, a significant role of *C. methylpentosum* in alleviating allergic airway inflammation could be postulated. Our data demonstrated that butyrate produced by gut microbiota modulation contributes to improving allergic airway inflammation. Still, the mechanism by which butyrate modulates the immune system has not been studied yet. Previously, the induction of regulatory T cells and their cytokines has been implicated as a major pathway for the regulation of the immune system by butyrate-producing bacteria. According to Atarashi et al. (2011), oral inoculation of *Clostridium* strains promoted a regulatory T-cell population and related cytokines resulting in attenuation of the diseased state in the gut. Unlike the previous results, we could not detect the induction of regulatory cytokines in our investigation (data not shown).

Thereby, we assumed the bacterial metabolite(s) might induce advantageous responses not by directly targeting the immune system but rather by indirectly contacting other systems such as epithelial linings.

GTB1 improved not only unbalanced immune reactions but also mucus hyperproduction. The gene expression of MUC5AC and mouse homolog of CLCA1 was significantly decreased (Figure 2A), and histological analysis via PAS and anti-MUC5AC IHC confirmed the reduction of mucus hypersecretion (Figure 2B). On the other hand, the expression of tight junction proteins was not modulated by the target strain (Supplementary Figure S3); thus, we hypothesized that GTB1 may regulate mucus production. As our results have shown, butyrate may be responsible for eliciting the ameliorative effects of asthmatic symptoms, and therefore, the levels of SCFA uptake receptors, GPRs, were measured. GPR41, GPR43, and GPR109a are well-known SCFA uptake receptors, and the specificity and potency of ligands are all different (Li et al., 2018). Supplementation of SCFAs changes the expression of GPRs and influences the microbiota, further resulting in attenuation of the diseased state (Lu et al., 2016). Specifically, GPR109a is essential for butyrate-mediated beneficial reactions, and GPR109a signaling can promote anti-inflammatory reactions (Singh et al., 2014). We found that oral administration of GTB1 significantly upregulated the gene expression of GPR109a in the lungs (Figure 6). These data proposed that the lungs as the inflamed target site of this model may be the main site for absorption of increased butyrate levels in the gut.

The direct effect of butyrate on the lungs is only sparsely demonstrated so far. However, it has been reported that butyrate can modulate mucin gene expression including MUC5AC, the mucin marker in our disease model, in human colonic goblet cells (Gaudier et al., 2004). However, studies dealing with the relation between MUC5AC, butyrate, and airway epithelial cells have not been conducted so far. To define the exact effect of butyrate on MUC5AC expression in the lungs, we used the human airway epithelial cell line, A549, based on a previous study with some modifications (Lin and Huang, 2020). We also investigated the potential for butyrate to exert toxic effects on A549 cells, which could potentially affect our assessment of its influence on MUC5AC expression. To address this concern, we conducted an MTT assay to verify A549 cell viability. The results showed no significant difference in cell viability between the media control group and the group treated with butyrate. *In vivo*, the pH of the nasal mucosa typically ranges from 5.6 to 6.7, while in the bronchia, it tends to be approximately 7.0 (Zajac et al., 2021). These findings challenge the definitive categorization of the respiratory tract as an acidic environment and raise questions about the potential impact of butyrate. This perspective gains support from studies indicating that butyrate can reach peripheral organs (Corrêa et al., 2022) and its detection in sputum and BALF samples (Ghorbani et al., 2015; Yue et al., 2020). Furthermore, the expression of butyrate receptors in the lungs, as provided in Figure 6, underscores the significance of butyrate’s association with the pulmonary tract. However, considering that most SCFAs, including butyrate, are primarily utilized for energy metabolism in the intestines, with only a minimal quantity reaching peripheral organs, it is unlikely that they would significantly alter the acidity within these organs. We took into account the real levels of butyrate in humans to determine its concentration. Reported butyrate concentrations in the lungs vary widely, ranging from 0 to 3 μM/g in the lung tissue of mice to 30 to 10,000 μM in the BALF or sputum samples from humans (Machado et al., 2021). Previous research has indicated that low concentrations of butyrate do not exhibit toxicity toward human airway cells (Konyalilar et al., 2019). Consequently, we conducted a dose–response study to identify the optimal butyrate dose and confirmed the absence of any adverse effects on cell viability with two specific doses (0.5 and 2 mM). After co-incubation with butyrate and A549 cells, MUC5AC mRNA expression levels were decreased in a dose-dependent manner (Figure 7), clearly proving the influence of butyrate on the regulation of MUC5AC gene expression. These data demonstrate that butyrate itself can inhibit the MUC5AC gene expression in airway epithelial cells and may play an essential role in the inhibition of mucus hypersecretion. The correlation between three factors (butyrate, MUC5AC, and airway epithelial cell) was explored in this research for the first time, conferring the novelty of this project.

We demonstrated that oral administration of GTB1 enhances butyrate production by gut microbiota modulation and inhibits mucin hypersecretion, resulting in alleviation of the diseased state in a murine allergic airway inflammation model. Still, the exact underlying mechanism of immune modulation is not fully understood. As mentioned earlier, we could not detect any significant induction of the regulatory cytokine in the GTB1-treated group. Subsequently, we measured the expression level of transcription factors of each T-cell subset, but clear differences were not found (data not shown). To confirm the exact responsible immune factor for these reactions, the immune cell population needs to be assessed further.

In conclusion, the potential of probiotic application for alleviating allergic asthma has received increased attention recently, and *L. plantarum* appears to be one of the most promising candidates. GTB1, with previously proven safety and functionality, was applied and evaluated for its beneficial effects on allergic airway inflammation. This strain significantly suppressed type 2 inflammation and mucus hypersecretion and modulated gut microbiota, especially by increasing *Clostridium* cluster IV which may be mainly responsible for the increase in butyrate levels. Butyrate uptake receptor in the lungs was also upregulated indicating a closely relevant effect of butyrate on the target site. In an *in vitro* experiment, butyrate could inhibit the gene expression of MUC5AC directly when co-incubated with human airway epithelial cells, demonstrating its possible influence on mucus hypersecretion. This study highlights the potential of GTB1 as a probiotic for the respiratory tract.

## Data availability statement

The raw data of 16S rRNA gene amplicons generated in the study are deposited in NCBI database under accession number PRJNA1020386 (https://www.ncbi.nlm.nih.gov/sra/PRJNA1020386).

## Ethics statement

The animal study was approved by the Committee on the Ethics of Animal Experiments of the Handong Global University (Approval No., HGUIACUC20210518-002; Approval date, 18 May 2021). The study was conducted in accordance with the local legislation and institutional requirements.

## Author contributions

HK: Conceptualization, Data curation, Funding acquisition, Investigation, Methodology, Project administration, Resources, Supervision, Writing – review & editing. H-SK: Conceptualization, Data curation, Formal analysis, Investigation, Methodology, Visualization, Writing – original draft, Writing – review & editing. BK: Conceptualization, Data curation, Investigation, Writing – review & editing. WH: Funding acquisition, Supervision, Writing – review & editing.
