# Supplementary material for: Lactiplantibacillusplantarum APsulloc331261 (GTB1™) promotes butyrate production to suppress mucin hypersecretion in a murine allergic airway inflammation model
Source: Front Microbiol. 2024 Feb 21;14:1292266. doi: 10.3389/fmicb.2023.1292266 (PMC10915089; doi:10.3389/fmicb.2023.1292266)
Supplement: Supplementary file 1 [file Data_Sheet_1.docx]

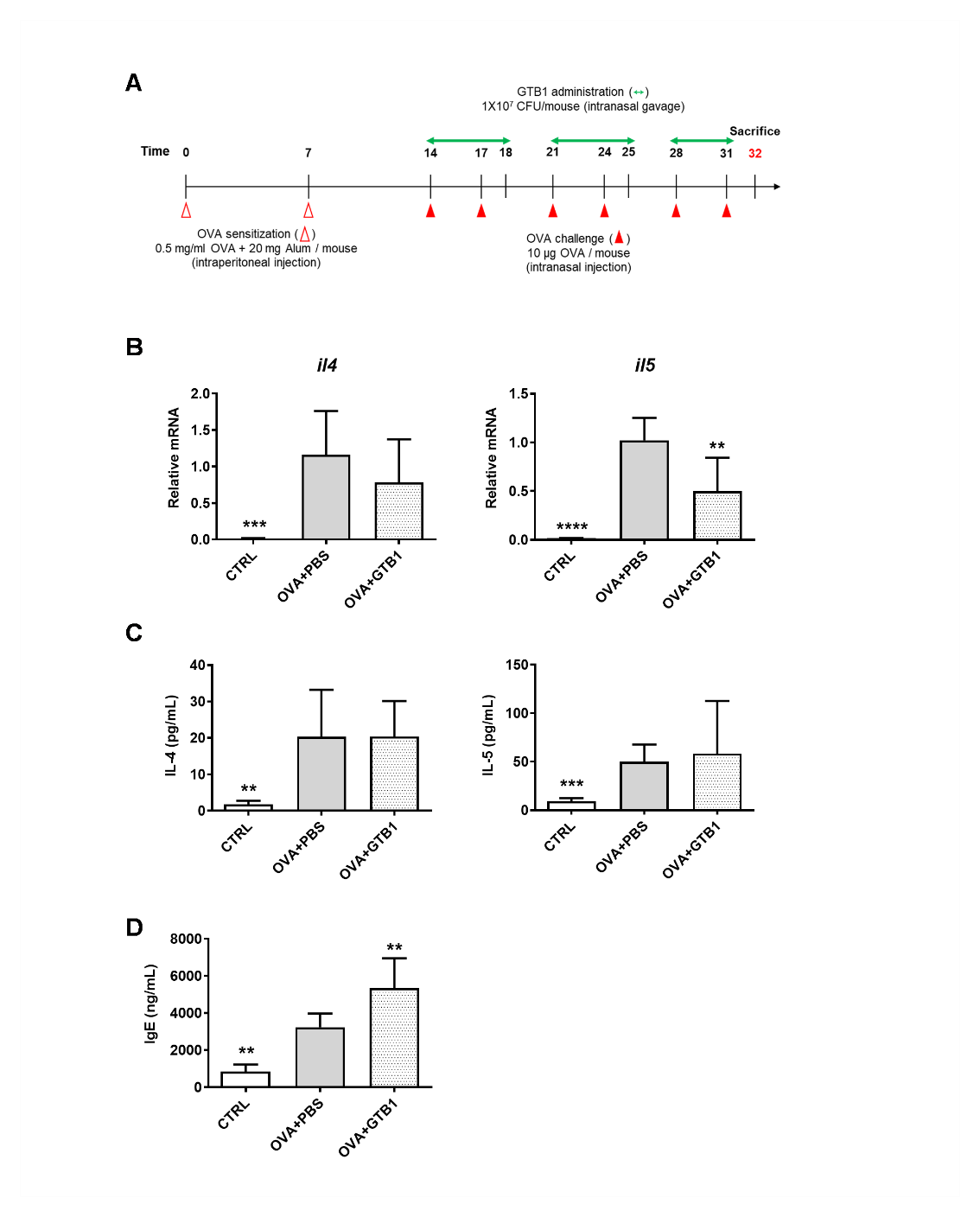


**Supplementary Figure 1.** Effects of intranasal administration of GTB1 on type 2 inflammation in an OVA-induced allergic airway inflammation murine model. (A) Scheme of the *in vivo* experiment, (B) lung mRNA expression levels of type 2 cytokines, (C) type 2 cytokines in BALF and (D) total IgE level in serum. *il4*: IL-4; *il5*: IL-5; CTRL: control group treated with vehicle only; OVA + PBS: OVA + vehicle treated group; OVA + GTB1: OVA + GTB1 intranasally treated group. Data show the mean ± SD (n = 4-5 mice per group); the difference compared to the OVA + PBS group was analyzed by one-way ANOVA with Fisher’s LSD test. **p*<0.05, ***p*< 0.01, ****p*< 0.001, *****p*< 0.0001.

**
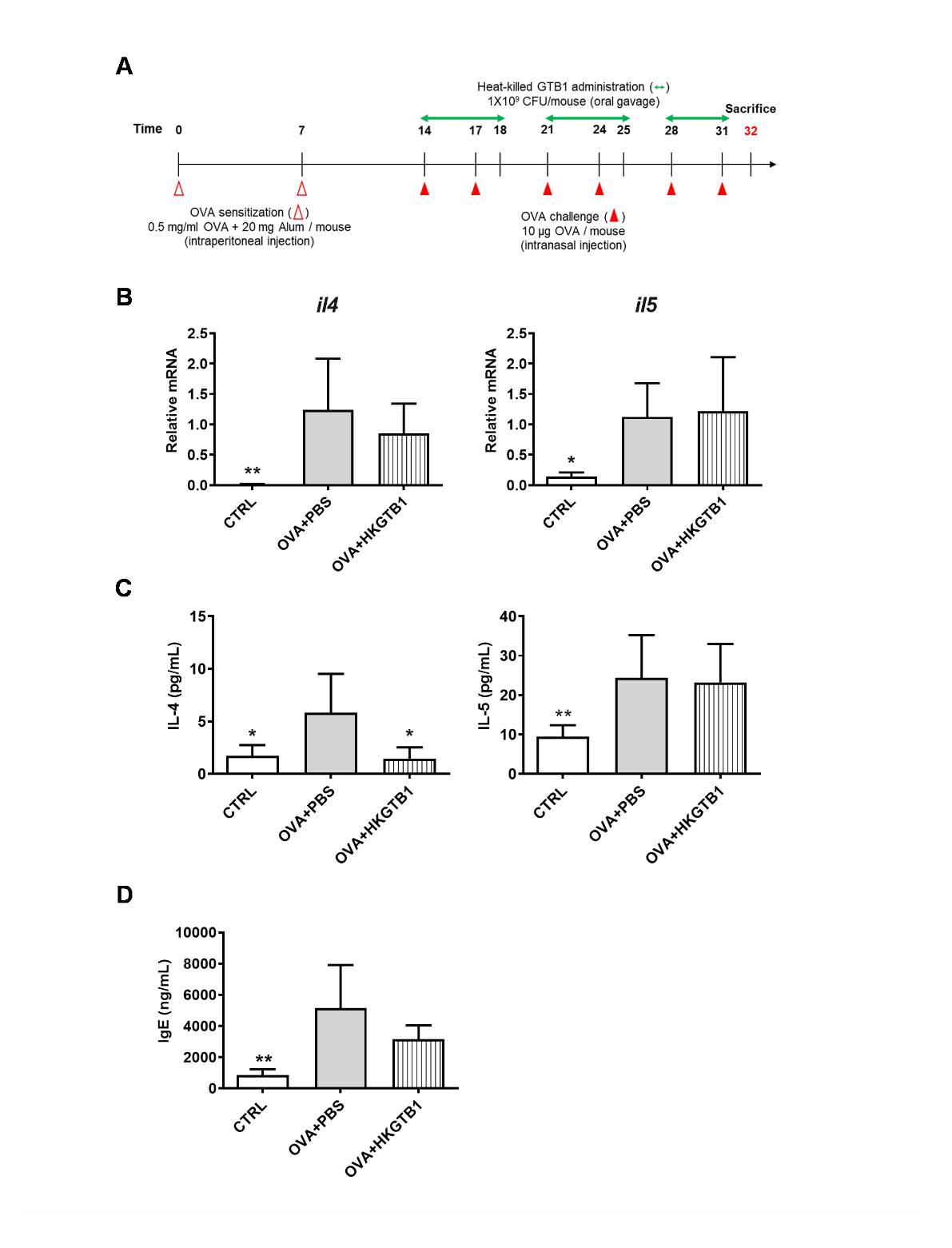
**

**Supplementary Figure 2.** Effects of oral administration of heat-killed GTB1 on type 2 inflammation in an OVA-induced allergic airway inflammation murine model. (A) Scheme of the *in vivo* experiment, (B) lung mRNA expression levels of type 2 cytokines, (C) type 2 cytokines in BALF and (D) total IgE level in serum. *il4*: IL-4; *il5*: IL-5; CTRL: control group treated with vehicle only; OVA + PBS: OVA + vehicle treated group; OVA + HKGTB1: OVA + heat-killed GTB1 treated group. Data show the mean ± SD (n = 5-7 mice per group); the difference compared to the OVA + PBS group was analyzed by one-way ANOVA with Fisher’s LSD test. **p*<0.05, ***p*<0.01.

**
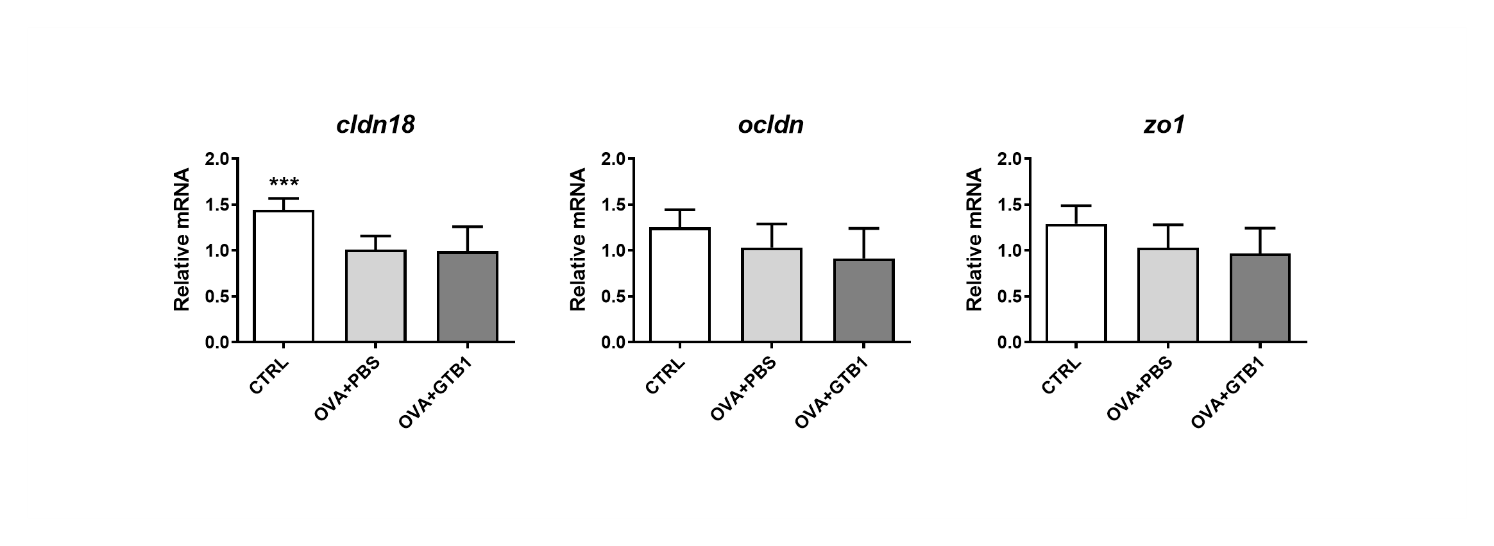
**

**Supplementary Figure 3.** Effects of oral administration of GTB1 on the expression of the epithelial tight junction proteins in an OVA-induced allergic airway inflammation murine model. *cldn18*: claudin-18, *ocldn*: occludin; *zo1*: zonula occludens-1; CTRL: control group treated with vehicle only; OVA + PBS: OVA + vehicle treated group; OVA + GTB1: OVA + GTB1 treated group. Data show the mean ± SD (n = 6-7 mice per group); the difference compared to the OVA + PBS group was analyzed by one-way ANOVA with Fisher’s LSD test.

**
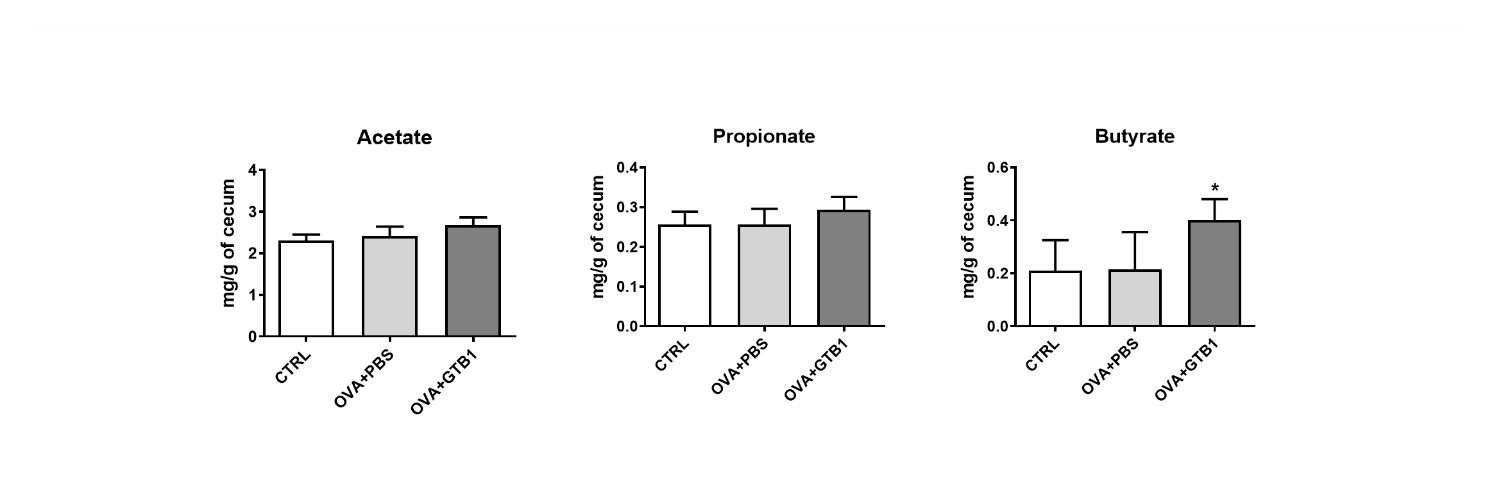
**

**Supplementary Figure 4.** Effects of oral administration of GTB1 on cecal SCFA production in an OVA-induced allergic airway inflammation murine model. CTRL: control group treated with vehicle only; OVA + PBS: OVA + vehicle treated group; OVA + GTB1: OVA + GTB1 treated group. Data show the mean ± SD (n = 3-6 mice per group); the difference compared to the OVA + PBS group was analyzed by one-way ANOVA with Fisher’s LSD test. **p*<0.05.

**Supplementary Table 1.** List of primers used for qRT-PCR

| **Target** | **Gene** | **Primer sequence (5’-3’)** | **Reference** |
| --- | --- | --- | --- |
| Mouse |  |  |  |
| Lung | *Il4* | F: GGC ATT TTG AAC GAG GTC AC | Shibata *et al.* (2018) |
|  |  | R: AAA TAT GCG AAG CAC CTT GG |  |
|  | *Il5* | F: CTC TGT TGA CAA GCA ATG AGA CG | Singh *et al.* (2017) |
|  |  | R: TCT TCA GTA TGT CTA GCC CCT G |  |
|  | *Ccl11* | F: GAA TCA CCA ACA ACA GAT GCA C | Yi *et al.* (2018) |
|  |  | R: ATC CTG GAC CCA CTT CTT CTT |  |
|  | *Ccl24* | F: ATT CTG TGA CCA TCC CCT CAT |  |
|  |  | R: TGT ATG TGC CTC TGA ACC CAC |  |
|  | *Muc5ac* | F: TCC CTT ACC TAA CCA GCA GAA | Tetaert *et al.* (2007) |
|  |  | R: GGG AGT ACA TGG AGA TGC TGT |  |
|  | *Muc5b* | F: GCA CGT AAA TGC GAC TGT CT |  |
|  |  | R: ATGGACCTTGCTCTCCTGAC |  |
|  | *Clca1* | F: AAT GGA TGA ATG GCT CAG TGA T | Huang *et al.* (2017) |
|  |  | R: TAT TGT AGG AGG ATG CGT TGT C |  |
|  | *Cldn18* | F: GAC CGT TCA GAC CAG GTA CA | Yang *et al.* (2019) |
|  |  | R: GCG ATG CAC ATC ATC ACT C |  |
|  | *Ocldn* | F: CCT CCA CCC CCA TCT GAC TA | Klaßen *et al.* (2017) |
|  |  | R: CTT CAG GCA CCA GAG GTG TT |  |
|  | *Zo1* | F: GCC GCT AAG AGC ACA GCA A | Chiaro *et al.* (2017) |
|  |  | R: TCC CCA CTC TGA AAA TGA GGA |  |
|  | *Gpr41* | F: CGA CGC CCA GTG GCT GTG GAC TTA | Samuel *et al.* (2008) |
|  |  | R: GTA CCA CAG TGG ATA GGC CAC GC |  |
|  | *Gpr43* | F: TTC TTA CTG GGC TCC CTG CC | Dewulf *et al.* (2011) |
|  |  | R: TAC CAG CGG AAG TTG GAT GC |  |
|  | *Gpr109a* | F: ATG GCG AGG CAT ATC TGT GTA GCA | Singh *et al.* (2014) |
|  |  | R: TCC TGC CTG AGC AGA ACA AGA TGA |  |
|  | *Actb* | F: GGC TGT ATT CCC CTC CAT CG | Shibata *et al.* (2018) |
|  |  | R: CCA GTT GGT AAC AAA TGC CAT GT |  |
| Human |  |  |  |
| Lung | MUC5AC | F: TCC ACC ATA TAC CGC CAC AGA | Yu *et al.* (2010) |
|  |  | R: TGG ACC GAC AGT CAC TGT CAA |  |
|  | ACTB | F: AGA AAA TCT GGC ACC ACA CC | Marcet *et al.* (2007) |
|  |  | R: GGG GTG TTG AAG GTC TCA AA |  |
